# Immediate assessment of performance of medical laboratory scientists following a 10-day malaria microscopy training programme in Nigeria

**DOI:** 10.1186/s41256-017-0051-x

**Published:** 2017-11-06

**Authors:** Bolatito Aiyenigba, Abiodun Ojo, Adolor Aisiri, Justus Uzim, Oluwole Adeusi, Halima Mwenesi

**Affiliations:** 1 FHI360 Nigeria, Abuja, Nigeria; 2 Malaria Consortium Nigeria, Abuja, Nigeria; 3 FHI360 Washington DC, Durham, USA

**Keywords:** Malaria, Microscopy, Training, Nigeria

## Abstract

**Background:**

Rapid and precise diagnosis of malaria is an essential element in effective case management and control of malaria. Malaria microscopy is used as the gold standard for malaria diagnosis, however results remain poor as positivity rate in Nigeria is consistently over 90%. The United States President’s Malaria Initiative (PMI) through the Malaria Action Program for States (MAPS) supported selected states in Nigeria to build capacity for malaria microscopy. This study demonstrates the effectiveness of in-service training on malaria microscopy amongst medical laboratory scientists.

**Method:**

The training was based on the World Health Organization (WHO) basic microscopy training manual. The 10-day training utilized a series of didactic lectures and examination of teaching slides using a CX 21 Olympus binocular microscope. All 108 medical laboratory scientists trained from 2012 to 2015 across five states in Nigeria supported by PMI were included in the study. Evaluation of the training using a pre-and post-test method was based on written test questions; reading photographic slide images of malaria parasites; and prepared slides.

**Result:**

There was a significant improvement in the mean written pre-and post-tests scores from 37.9% (95% CI 36.2–39.6%) to 70.7% (95% CI 68.4–73.1%) (*p* < 0.001). The mean counting post-test score improved significantly from 4.2% (95% CI 2.6–5.7%) to 27.9% (95% CI 25.3–30.5%) (*p* < 0.001). Mean post-test score for computer-based picture speciation test (63.0%) and picture detection test (89.2%) were significantly higher than the mean post-test score for slide reading speciation test (38.3%) and slide reading detection test (70.7%), *p* < 0.001 in both cases.

**Conclusion:**

Parasite detection and speciation using enhanced visual imaging was significantly improved compared with using direct microscopy. Regular in-service training and provision of functional and high resolution microscopes are needed to ensure quality routine malaria microscopy.

## Background

Malaria remains a global public health concern and a recent publication by the World Health Organization (WHO) estimates a world-wide malaria burden of 198 million with an estimated 584,000 deaths. Eighty-two per cent of the estimated cases and 90% estimated deaths are in Africa. Only two countries i.e. Nigeria and the Democratic Republic of Congo account for 34% of the global estimated cases and 39% estimated deaths. By 2015, these two countries accounted for 35% of malaria mortality in Sub-Saharan Africa [1].

Nigeria adopted the policy of parasite based diagnosis before treatment of suspected cases in 2007 to align with the WHO 3Ts initiative of test, treat and track. However, there were challenges to satisfactory implementation of the policy. Over-diagnosis and over-treatment of malaria persist despite large scale up of malaria rapid diagnostic tests across the country [2]. The National Guideline for Diagnosis and Treatment of Malaria in Nigeria (2015) [3] recommends the use of microscopy only in tertiary and secondary health facilities where there are functional laboratory services with qualified laboratory scientists. Malaria rapid diagnostic tests (mRDTs) are recommended for use in primary health care settings where there are no qualified laboratory scientists. However, the quality of malaria microscopy is still a concern despite its gold standard status. Various studies across Africa and in Nigeria have reported misdiagnosis with microscopy; mainly over diagnosis of malaria which invariably leads to over treatment of persons with false positive results [4–9]. The implication of this is that the burden of malaria is exaggerated while consumption of Artemisinin Combination Therapy (ACT) is very high and mainly based on false positive diagnosis.

Several reasons have been documented for the poor malaria microscopy diagnosis. These include insufficient skills of medical laboratory scientists; high work burden and/or poorly motivated laboratory scientists; poorly equipped parasitological laboratories - lack of good microscopes and reagents, − and lack of supportive supervision [10], [11].

Various studies on the other hand have demonstrated the effectiveness of in-service training on malaria microscopy diagnosis, which has been reported to improve the performance of medical laboratory scientists [12–19], however, available documented information in Nigeria is scanty despite a lot of work having being done in this area. This paper seeks to document the MAPS project’s experience in building capacity of medical laboratory scientists to improve malaria diagnosis across various geographical locations in Nigeria using training data routinely collected. A series of 10-day microscopy training courses using the WHO curriculum were undertaken by the Family Health International (FHI) 360 Malaria Action Program for States (MAPS) project in collaboration with the United States Department of Defence/Walter Reed project. This training was necessary to provide a pool of very good malaria microscopists across geographic locations where the project implemented activities. These trained scientists will provide the needed on-the-job training of other scientists, provide oversight and build confidence of clinicians on malaria microscopy and diagnosis in general.

In a largely populated country like Nigeria, the options were limited as the medical professionals were not convinced about the wide scale use of malaria rapid diagnostic tests and repeat malaria microscopy test were coming up as positive (though these were false positive).

The performance of the trained medical laboratory scientists was evaluated before and immediately after the training. The study did not follow participants to their facilities nor examine facility data before the training and after the training. The focus was on the immediate training outcome. MAPS was a 6-year (2010–2016) United States’ President’s Malaria Initiative (PMI) funded malaria project that supported the National Malaria Elimination Programme (NMEP) in scaling up proven malaria control strategies including strengthening malaria parasite based diagnosis at all levels of the health care system. The United States Department of Defence/Walter Reed project was responsible for providing the facility (venue) and trainers for the malaria microscopy training. MAPS was responsible for other training logistics including collaborating with the states laboratory services to ensure appropriate participants were selected, and other post-training follow up activities. As at the time of the implementation of the MAPS project, there were no sufficient trainers available across the country to consider alternative mode of training; such as a decentralised training plan or on-the-job training; which will meet the project timelines and deliverables. When sufficient pool of trainers and high quality microscopes are available, clustered training for continuous medical education of laboratory scientists and e-learning may be a cheaper alternative to the 10-day residential training.

## Methods

### Study sites

Public secondary and tertiary health facilities with laboratory scientists, facilities and infrastructure for conducting malaria microscopy in Ebonyi, Kogi, Cross River, Nasarawa and Zamfara States were included in the study. Secondary and tertiary level health facilities that had no laboratory scientists and infrastructure for malaria microscopy were excluded.

### Training materials

Training materials included a CX 21 Olympus binocular microscope, pH meter and malaria microscopy consumables. In addition, each participant received a hard copy of the course manual, laminated bench aids and a CD-ROM of *Plasmodium* parasites identification slides and slide preparation templates. The laminated bench aid is a pictorial representation of various plasmodium parasite images. It is used by each participant as a guide in identifying the plasmodium species on the microscopy slides. In addition, the CD-ROM contains similar images and it was given to each participant to use during the training. Both learning materials were designed to aid participants at work post-training period.

### Inclusion criteria for participant’s selection:

The selection of participants was carried out by the supervising government ministry and health management board overseeing government owned secondary health facilities across states where the MAPS project was being implemented. Participants were selected based on the following criteria.

1. A practicing medical laboratory scientist, registered with the Medical Laboratory Scientist Council of Nigeria,
2. A practicing medical laboratory scientist involved in routine malaria diagnosis in his/her facility,
3. Below grade level 12 in the civil service employment (therefore more likely to be practicing on the bench) and
4. Willing and available to participate fully in all training sessions.

The training was carried out between 2012 and 2015.

### Training intervention

#### Training goal:

The goal of the training was to provide required knowledge and skills to improve participants’ competency and proficiency in identifying malaria plasmodium species for making accurate malaria diagnosis using microscopy. Although slide preparation was taught during the training because the quality of slide may affect the identification of the parasite, the process of slide preparation was not measured and/or scored.

#### Trainers:

The trainers were qualified medical laboratory scientists who had been trained as trainers on malaria microscopy and were drawn from various tertiary institutions in the country. The MAPS project was only involved in the training logistics and ensuring participants remained on malaria diagnosis benches after training so learning could be utilized. Each session had 3 trainers to 15 participants. Although only one co-author was directly involved in coordinating the training, the other co-authors had responsibility for the impact of the training in the broader quality of malaria diagnosis and malaria service delivery in general.

#### Training process:

The 10-day standard malaria microscopy training was based on WHO basic microscopy learners guide. The training was a necessary requirement for all practicing medical laboratory scientists conducting malaria microscopy in secondary and tertiary health facilities in project supported facilities. It aimed to improve the quality of malaria microscopy services and reduce the difference between negative malaria diagnosis using mRDTs and false positive malaria microscopy tests witnessed in the facilities. Learning and skills enhancement were the key advantages of participation in the training. We expect that these advantages will lead to improved performance and related recognition for the health worker, as well better services for clients.

The training approach consisted of a series of didactic lectures, group presentations; problem based learning, Giemsa stain preparation, power point presentations on implementation issues related to malaria blood film preparation, staining of blood films with Giemsa stain, and examination of high quality teaching stained slides for the major *Plasmodium* species. In addition, lectures on slide reading and counting basics were repeated each morning between the 3rd and 10th day of the training to ensure participants understood the fundamentals of Plasmodium species detection, staging and speciation.

Also, continuous assessment of all participants was conducted from Day 3 to Day 8; this assessment was conducted with 5 to 8 slides during the practical session to assess the performance of the participants and to ensure that participants covered all aspects of the standard training curriculum. Any knowledge gaps were identified and resolved during the training. The training was conducted over two weeks (10 working days) for 8 h per day for a total 80 training hours. All participants completed the 80 h training session.

### Measurement of training outcome

Based on the Blooms taxonomy, the learning outcome for this training was intended to assess participant’s knowledge and skills-based functions. The questions for the written test and picture slide reading test were intended to assess participants’ knowledge of malaria microscopy diagnosis, capacity to understand training content, correct recall of malaria parasite slide images projected and correct assessment and identification of such slides.

The counting test, slide detection and speciation test was intended to assess participants’ skills in identifying malaria parasites using microscopy and in counting detected malaria parasites. Microscopy slide preparation was not formally assessed and scored in this training. All test scores were calculated based on percentage of right answers to questions and each session had a maximum score of 100%.

Overall, the training process was assessed using a pre-and post-test evaluation method, the immediate outcome of the training intervention was assessed based on three criteria: written test, computer-based picture test and malaria microscopy slide reading tests.

### Written test

The written test comprised of a set of 30 instructive multiple choice questions to test participant’s knowledge on malaria microscopy before and after training.

### Computer-based picture test

The computer-based test comprised of 30 magnified photographic snapshots of quality slides with images of malaria parasites.

### Slide reading test

**Fig. 1:**
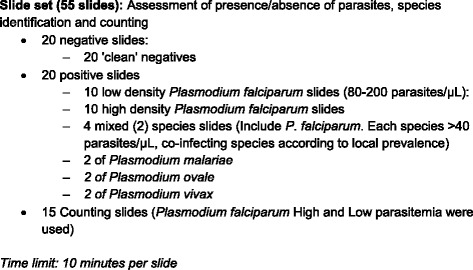
WHO standard slide panel used for assessment

A total of 55 quality pre-stained slides by a WHO certified malaria microscopists were used to assess participant’s skills in detection, speciation and counting of malaria parasites (Fig. 1).

### Data management and statistical analysis

This is a retrospective study. Test scores and participants’ registration data routinely collected during training were extracted using Microsoft Office Excel® 2010 template; data was cleaned and exported to Stata 11, StataCorp. 2009. *Stata Statistical Software: Release 11*. College Station, TX: StataCorp LP for data analysis. To assess the performance after the training intervention, paired-test was used to determine if there was any significant difference between the performance scores before and after the training. Statistical significance level was set at 0.05 and a two-tailed paired t-test was conducted to compare pre-and post-test mean scores.

## Results

Data from all the 108 medical laboratory scientists trained between 2012 and 2015 was analysed; majority were males 76 (70.4%); 100 (92.6%) were from secondary health facilities; and 96% were on WHO expert level 4 (WHO expert level is a competency based level used to categorize malaria microscopists based on their performance in correctly identifying malaria parasites during assessment. Microscopists are categorized into four levels following a competency assessment based on WHO curriculum, with level 1 being the highest skilled level) (Table 1).

**Table 1:** Distribution of general characteristics of medical laboratory scientist trained on malaria microscopy

Characteristics		Frequency *n* = 108 (%)
Sex	Male	76 (70.4)
	Female	32 (29.6)
Type of health facility	Secondary	100 (92.6)
	Tertiary	8 (7.4)
State	Cross River	29 (26.9)
	Ebonyi	10 (9.3)
	Kogi	29 (26.9)
	Nasarawa	18 (16.7)
	Zamfara	22 (20.4)
*WHO Expert level	Level 1	0 (0.0)
	Level 2	1 (0.9)
	Level 3	3 (2.8)
	Level 4	104 (96.3)

*Microscopists are categorized into four levels following a competency assessment implemented/supervised by WHO and based on WHO curriculum, level 1 is the highest skilled level

Data from pre-and post-training assessments showed significant improvement on all parameters used. Table 2 detailed the improvement on the five components of slide reading; across the four components of the computer-based tests; and the written test.

**Table 2:** Results of test of significance of mean pre-and post-test scores using paired sample t-test

Category of test	Pre-test		Post-test		*P*-value
	Mean (%)	95% CI	Mean	(95% CI)	
Slide Reading Tests					
Detection	53.9	51.4–56.4	70.7	67.8–73.6	<0.001
Speciation	18.9	16.0–21.7	38.3	34.5–42.0	<0.001
Specificity Test	45.7	40.9–50.5	62.4	57.6–67.2	<0.001
Sensitivity Test	60.9	56.7–65.0	78.4	75.4–81.5	<0.001
Counting Test	4.2	2.6–5.7	27.9	25.3–30.5	<0.001
Computer-Based Tests					
Picture Detection	69.3	66.2–72.4	89.2	87.3–91.1	<0.001
Picture Speciation	24.6	21.2–28.0	63.0	60.0–66.1	<0.001
Picture Staging	33.4	29.6–37.3	73.5	71.0–76.1	<0.001
Written Test					
Writing Test	37.9	36.2–39.6	70.7	68.4–73.1	<0.001

The results also showed greater improvement in post-tests scores for computer-based picture speciation test when compared with slide reading speciation test. (*P* < 0.05) Table 3.

**Table 3:** Results of test of significance using paired t-test to compare Speciation Tests for slide reading and computer based picture pre-and post-tests

Category of test	Mean test score (95% CI)	*P*-value
Slide reading Speciation pre-test	18.9 (16.0–21.7%)	0.10
Computer based picture Speciation pre-test	24.6 (21.2–28.0%)	
Slide reading Speciation post-test	38.3 (34.5–42.0%)	<0.001
Computer based picture Speciation post-test	63.0 (60.0–66.1%)	

Table 4 shows statistical significant difference in both pre-and post-tests scores for picture detection tests when compared with slide reading detection tests (*P* < 0.05).

**Table 4:** Results of test of significance using paired t-test to compare Detection Tests for Slide reading and Computer based picture pre-and post-tests

Category of test	Mean test score (95% CI)	*P*-value
Slide reading detection pre-test	53.9 (51.4–56.5%)	<0.001
Computer based picture detection pre-test	69.3 (66.2–72.4%)	
Slide reading detection post-test	70.7 (67.8–73.6%)	<0.001
Computer based picture detection post-test	89.2 (87.3–91.1%)	

## Discussion

In this study, we have described the effectiveness of a 10-day malaria microscopy training to improve the diagnostic capacity of trained medical laboratory scientist across five states in Nigeria. The training was conducted as part of the efforts to improve malaria microscopy diagnosis among laboratory scientists in addition to the widespread deployment and training of other health workers on rapid malaria diagnosis. The trainees’ performances were assessed using written, slide reading and computer based picture tests. Overall, the training programme led to an improvement in the post-test performance of participants’ malaria diagnostic knowledge and ability to interpret real malaria stained slides and computer based picture slides, which is consistent with results from other studies [13–16]. However, because the data used for this paper was collected as part of routine project implementation, there were insufficient data such as age, educational level of participants, the number of years in service and previous related in-service training which may affect training performance and act as confounder in the immediate outcome of the training. A similar study on Acid Fast Bacilli microscopy training found that those with diplomas and those who had attended similar training performed significantly better than those with degrees and those attending the training for the first time [13]. Another similar study conducted in Nigeria showed statistically significant differences in pre-test median score among the three categories of participants in picture test and basic malariology test only [20] though this study had only medical laboratory scientists as participants.

This study however shows that the malaria microscopy content in the current curriculum of formal academic laboratory training for medical laboratory scientists is not sufficient to make accurate malaria diagnosis in the field. Only four laboratory scientists out of a hundred and eight had been certified above level 4 in the WHO expert category, others were at level 4. This is grossly inadequate for a nation with a high malaria burden aiming to adequately control and progress to malaria elimination. Periodic in-service malaria microscopy training and perhaps the review of the malaria diagnosis content and methodology of training in the formal training curriculum for medical laboratory scientists may be necessary to assist in producing adequate numbers of laboratory scientists that will support Nigeria’s quest to control malaria and eventually join the league of nations on the road to malaria elimination. The cost of training laboratory scientists for 10-days off site is not sustainable, innovative ways such as e-learning and on-site training are required using the already trained pool of scientists to sustain malaria microscopy skills.

Unlike other similar training interventions in Nigeria [20], the MAPS project focused malaria microscopy training for laboratory scientists alone, and strengthened the use of malaria rapid diagnostic test, which required minimal skill, among other categories of health workers. In addition, the laboratory scientists were trained on the malaria rapid diagnosis, convinced on its effectiveness and persuaded to allow other categories of health workers make diagnosis of malaria in their absence using the rapid test. This was done to ensure that patients were correctly diagnosed, therefore correctly treated, lowering transmission – leading to lowering of morbidity and mortality, and firmly putting Nigeria on the path to effective control of malaria.

The result of analysis from this study showed very low mean pre-test scores on counting (parasite density) and species identification (speciation) as shown in Table 2 **,** when compared with other pre-test scores. A similar pattern was reported in a recent study by Olukosi et al.; in Nigeria [20]. In that study, counting test scores increased from 0 to 25% while in this study it increased from 4.2% to 27.9%; similarly, slide reading speciation test scores increased in that study by a margin of 11.3% compared to 19.4% in this study. Compared to parasite detection, it seems specie identification and counting were more difficult skills to acquire. Published studies have also shown that practicing medical laboratory scientist find it difficult to differentiate malaria species under the microscope [21], [22].

Speciation and quantification of malaria parasites are important skills in monitoring disease severity and drug efficacy hence the findings from this study imply the need to tailor future trainings and mentoring programmes to focus on identification of malaria species, differentiating species from artefacts and quantifying parasites [23].

Despite the initial poor performance in the ability of trainees to identify species of malaria parasite, the post training performance based on computer picture tests was better when compared to specie identification using stained slides. The computer-based picture detection tests score was also higher than the slide reading detection tests score for both pre-and post-tests. Accurate malaria microscopy diagnosis in real-life is based on the use of a high-resolution microscope, a well-prepared smear and quality staining of smear slides. In this study, the slides were stained and prepared by experienced and expert microscopists for the trainees’ pre-and post-assessment. This suggests that medical laboratory scientists in Nigeria and other developing countries may require the magnification of the malaria parasite images beyond what the resolution of the routinely used microscopes provides. Some studies have shown promising results with the use of low cost technology to improve image magnification and clarity [23–29]. This is an indication for further research into more cost-effective and simple to use technology that will enhance the quality of malaria parasite images viewed with standard microscopes. External Quality Assurance (EQA) implemented after the training activities showed that provision of functional high resolution microscopes and laboratory consumables such as slides (to prevent recycling), and good quality reagents are needed to achieve quality malaria diagnosis using microscopy. National malaria control programs in malaria endemic countries need to articulate the needs beyond individual capacity building to institutional strengthening and guide donors likewise.

The drive for accurate diagnosis of malaria in effective control of malaria in Nigeria needs to go beyond in-service training of personnel to the training institutions. Modern training technology such as computer-based pictures used by implementing partners funded by the United States Government should be provided in the nation’s training institutions. As the campaign for malaria parasitological diagnosis before treatment increases, the confidence of clinicians in the gold standard needs to be restored through regular capacity building, supportive supervision and infrastructural upgrade. Many participants went back to their hospitals and were forced to continue using their sub-standard old microscopes which reduced the accuracy of their diagnosis. Health facilities should be supported to upgrade their laboratory facilities for effective use of knowledge and skills acquired during training by their staff.

This study was not without limitations. Some characteristics of the participants may have confounded the results but these data were not available. Another limitation was the fact that it was assumed that the laboratory scientists should know how to prepare good quality slides for malaria microscopy so slide preparation was not assessed and documented. The study in addition, only assessed immediate results of the training. Medium to long term assessment was not conducted to ascertain how the training materials were used after the training; and what knowledge and skills were retained post training. Hence, we may not be able to tell if the immediate post training results were sustained months to years after the training; and the percentage of trained personnel that were retained by their employer to continue malaria microscopy. However, the MAPS project supported the states to institute supportive supervision including external quality assurance for malaria diagnosis, the data generated from that support will better reflect the medium-term impact of the training.

## Conclusion

The study demonstrated evidence of improved malaria diagnostic performance following the WHO standard 10-day malaria microscopy training. To sustain and improve on the outcome of this training, periodic refresher training should be planned for; and routine external quality assurance process established to follow up and mentor these trained medical laboratory scientists. In addition, there is need for further research to evaluate the use of low cost technologies in enhancing malaria microscopy images including strengthening of the quality of malaria microscopy during pre-service training in all relevant institutions.

## Abbreviations

ACTs: Artemisinin Combination Therapy
AFB: Acid Fast Bacilli
MAPS: Malaria Action Program for States
MLS: Medical Laboratory Scientist
mRDT: malaria Rapid Diagnostic Tests
PMI: President’s Malaria Initiative
USAID: United States Agency for International Development
WHO: World Health Organization
